# Baseline Survey of JPHC Study – Design and Participation Rate

**DOI:** 10.2188/jea.11.6sup_24

**Published:** 2007-11-30

**Authors:** Shoichiro Tsugane, Tomotaka Sobue

**Affiliations:** 1 Epidemiology and Biostatistics Division, National Cancer Center Research Institute East.; 2 Cancer Information and Epidemiology Division, National Cancer Center Research Institute.

**Keywords:** baseline survey, questionnaire, blood, health check-up data

## Abstract

The data collection from cohort subjects at baseline is the core work for prospective study as well as follow-up. We set up 140,420 cohort subjects (68,722 men and 71,698 women) (61,595 in 1990 as Cohort I and 78,825 in 1993 as Cohort II) based on resident registry of 29 districts under 11 Public Health Center areas and baseline survey were submitted for them. The survey consisted of the following three components: (1) self-administered questionnaire survey, (2) collection of blood samples (plasma and buffy coat) for deep-freezed storage and (3) collection of health check-up data. All survey were completed during the first five year of each study. Among all cohort subjects, 113,461 (81%) (53,375 men and 60,086 women, 50,245 in Cohort I and 63,216 in Cohort II) returned the questionnaire and 49,011 (35%) (18,159 men and 30,852 women, 24,637 in Cohort I and 24,374 in Cohort II) provide their blood. The health check-up data were collected from 47,910 (34%) (17,276 men and 30,664 women, 23,311 in Cohort I and 24,599 in Cohort II). These data and blood samples serve as basis for the Japan Public Health Center-based prospective Study on cancer and cardiovascular diseases (JPHC Study).

## INTRODUCTION

The data collection from cohort subjects at baseline is the core work for prospective study as well as follow-up. The data obtained from questionnaire is usually served as baseline data in most prospective studies. However these data are subjective and can be unreliable in some factors. More objective information is useful and thus human materials such as blood, tissue and toenail are frequently collected and stored for future use in prospective studies.

## METHODS

The Japan Public Health Center-based prospective Study on cancer and cardiovascular diseases (JPHC Study) was initiated by 4 population cohorts and a health checkup cohort (Cohort I) in 1990 and it merged 5 population cohorts and two Suita city cohorts (Cohort II) in 1993.

### Subjects

#### Cohort I

As of January 1, 1990, we established a population-based cohort of 54,498 residents (27,063 men and 27,435 women) who registered their address in 14 administrative districts (city, town or village) supervised by four Public Health Center (PHC) areas: 12,291 from Ninohe city and Karumai town in Ninohe PHC area, Iwate prefecture, 15,782 from Yokote city and Omonogawa town in Yokote PHC area, Akita prefecture, 12,219 from Usuda, Saku, Koumi, Kawakami towns and Yachiho, Minami-aiki, Kita-aiki, Minami-maki villages in Saku PHC areas, Nagano prefecture, 14,206 from Gushikawa city and Onna village in Ishikawa PHC area, Okinawa prefecture, and who was born from January 1, 1930 to December 31, 1949 (40 to 59 years of age). We further included 7,097 participants (2,919 men and 4,178 women) of health checkup program in Katshushika PHC area in Tokyo metropolis from the fiscal year 1990 to 1994 (2,440 in 1990, 2,211 in 1991, 173 in 1992, 1,033 in 1993, and 1,240 in 1994), in which all residents with 40 and 50 year-old were invited. Five PHC areas were selected based on variation in mortality rate of stomach cancer for our previous ecological study, in which randomly selected subjects were intensively examined^1^^, ^^2^^, ^^3^^)^.

#### Cohort II

As of January 1, 1993, we established a population-based cohort of 62,398 residents (30,651 men and 31,747 women) who registered their address in 13 administrative districts (city, town or village) supervised by five PHC areas: 21,488 from Tomobe town and Iwase town in Kasama PHC area, Ibaraki prefecture, 3,571 from Oguni town in Kashiwazaki PHC area, Niigata prefecture, 8,606 from Kagami town and Noichi town in Tosayamada PHC area, Kochi prefecture, 14,624 from Uku town, Ojika town, Shin-uonome town, Arikawa town, Kamigoto town and Narao town in Arikawa PHC area, Nagasaki prefecture, and 14,109 from Hirara city and Gusukube town in Miyako PHC area, Okinawa prefecture, and who was born from January 1, 1923 to December 31, 1952. In Suita city in Suita PHC area, Osaka prefecture, two different cohorts were set up. The first cohort (Suita 1) was defined as all 9,747 residents (4,793 men and 4,954 women) in Suita city with 40 or 50 year-old in the fiscal year 1993, because they were invited to the comprehensive health checkup program conducted by the city. The second cohort (Suita 2) was defined as a part of the Suita study^4^^)^, in which subjects were arbitrarily selected based on the population registry of the city, in the years 1989 through 1992 and aged 30 to 79 years, stratified by sex and 10 year age group. The 6,680 subjects (3,296 men and 3,384 women) with aged 40 to 69 years as of April 1, 1993 were used for the JPHC study. Six PHC areas were selected considering geographical distribution and feasibility.

### Baseline Survey

*Questionnaire*: A self-administered questionnaire was submitted to all cohort subjects and asked to report their lifestyle such as socio-demographic situation, personal medical history, smoking and drinking history, and dietary habits. The questionnaire used in Cohort II was modified in several items. The questionnaire was distributed mostly by hand or partly by mail in 1990 (as a rule) in 4 populations in Cohort I and in 1993 (as a rule) in 5 populations in Cohort II. The incomplete answer was supplemented by telephone interview. In Katsushika PHC area, questionnaire was interviewed on the occasion of health checkup from 1990 to 1994. In Suita PHC area, the questionnaire was mailed to all the subjects from 1993 to 1995 and supplemented by interview for participants of health checkup on that occasion or by telephone interview for those who did not attend the health check-up.

*Blood and health checkup*: A total of 10 ml blood was provided voluntarily by cohort subjects and gathered into heparinized tube in the occasion of health checkup program sponsored by each local government mostly or company in some cases. The purpose and human rights for special blood collection for JPHC Study was informed to all cohort subjects. The tube was centrifuged for 10 min at 3,500-4,000 rpm to obtain plasma and a buffy coat layer within 12 hours. The plasma and buffy layer were divided into four 1.0 ml tubes (three for plasma and one for buffy layer) and stored at -80°C. The blood was collected in 1990 to 1992 (1990 to 1994 for Katsushika PHC cohort) in Cohort I and from 1993 to 1995 in Cohort II. The data from the health checkup was also obtained in this occasion. The common items are the following: anthropometrical measures (height and weight), blood pressures (systolic and diastolic), urinalysis (protein, sugar and blood in spot urine), lipids (total cholesterol, HDL-cholesterol, trygliceride), liver function test (GOT or AST, GPT or ALT and gamma-GTP), anemia test (red blood cell count, haematcrit and hemoglobin), blood sugar, uric acid and hepatitis B virus surface antigen (HBsAg).

## RESULTS

### Cohort I (Table 1a,b,c)

﻿﻿Among 54,498 population-based cohort subjects and 7,097 health checkup cohort subjects, 43,149 (79%), 20,665 (76%) men and 22,484 (82%) women, and 7,096 (100%) (2,919 men and 4,177 women) returned their questionnaires, respectively. Although date of entry was distributed from January 1990 to May 1992 in 4 population-based cohort, 54% was concentrated between February 1990 and March 1990. Only 4% responded in 1991 or later. 17,587 (32%) population cohort, 6,556 (24%) men and 11,031 (40%) women, and 7,050 (99%) health checkup cohort, 2,901 men and 4,149 women, provided their blood and stored their plasma and buffy layer at -80. We also obtained health checkup data from 17,923 (33%) population cohort, 6,532 (24%) men and 11,391 (42%) women, and 5,388 (76%) health checkup cohort, 2,245 (77%) men and 3,143 (75%) women.

**Table 1a.** Participants of baseline survey, cohort I.

	Subjects	Questionnaire				Check-up data collected %		Blood provided %	

		returned %		analyzed %					
Ninohe, Iwate	12,291	9,510	(77)	9,101	(74)	5,044	(41)	5,120	(42)
Ninohe city	8,313	6,381	(77)	6,139	(74)	3,193	(38)	3,235	(39)
Karumai town	3,978	3,129	(79)	2,962	(75)	1,851	(47)	1,885	(48)
Yokote, Akita	15,782	12,002	(76)	11,754	(75)	5,165	(33)	4,608	(29)
Yokote city	12,383	8,802	(71)	8,623	(70)	3,346	(27)	2,980	(24)
Omonogawa town	3,399	3,200	(94)	3,131	(92)	1,819	(54)	1,628	(48)
Saku, Nagano	12,219	11,055	(91)	10,887	(89)	5,324	(44)	5,494	(45)
Usuda town	4,246	3,885	(92)	3,838	(90)	1,801	(42)	1,802	(42)
Saku town	2,338	2,052	(88)	2,010	(86)	834	(36)	966	(41)
Yachiho village	1,324	1,223	(92)	1,206	(91)	633	(48)	646	(49)
Koumi town	1,682	1,491	(89)	1,465	(87)	740	(44)	743	(44)
Minami-aiki village	382	339	(89)	334	(87)	207	(54)	208	(55)
Kita-aiki village	269	244	(91)	239	(89)	153	(57)	153	(57)
Minamimaki village	830	760	(92)	756	(91)	379	(46)	383	(46)
Kawakami town	1,148	1,061	(92)	1,039	(91)	577	(50)	593	(52)
Ishikawa, Okinawa	14,206	10,582	(75)	10,453	(74)	2,390	(17)	2,365	(17)
Gushikawa city	12,279	9,003	(73)	8,874	(72)	1,610	(13)	1,583	(13)
Onna village	1,927	1,579	(82)	1,579	(82)	780	(41)	782	(41)
Sum of 4 population cohorts	54,498	43,149	(79)	42,195	(77)	17,923	(33)	17,587	(32)
Katsushika, Tokyo*	7,097	7,096	(100)	7,004	(99)	5,388	(76)	7,050	(99)
1990 participants	2,440	2,440	(100)	2,423	(99)	1,555	(64)	2,423	(99)
1991 participants	2,211	2,210	(100)	2,193	(90)	1,388	(63)	2,206	(100)
1992 participants	173	173	(100)	170	(98)	173	(100)	170	(98)
1993 participants	1,033	1,033	(100)	993	(96)	1,032	(100)	1,022	(99)
1994 participants	1,240	1,240	(100)	1,225	(99)	1,240	(100)	1,229	(99)
Total	61,595	50,245	(82)	49,199	(80)	23,311	(38)	24,637	(40)

* Participants of health check-up program at 40 and 50 years of age

**Table 1b.** Participants of baseline survey, cohort I males.

	Subjects	Questionnaire				Check-up data collected %		Blood provided %	

		returned %		analyzed %					
Ninohe, Iwate	6,022	4,393	(73)	4,229	(70)	1,777	(30)	1,844	(31)
Ninohe city	4,054	2,923	(72)	2,825	(70)	1,109	(27)	1,153	(28)
Karumai town	1,968	1,470	(75)	1,404	(71)	668	(34)	691	(35)
Yokote, Akita	7,559	5,576	(74)	5,471	(72)	1,733	(23)	1,601	(21)
Yokote city	5,899	4,049	(69)	3,974	(67)	943	(16)	873	(15)
Omonogawa town	1,660	1,527	(92)	1,497	(90)	790	(48)	728	(44)
Saku, Nagano	6,173	5,479	(89)	5,410	(88)	2,222	(36)	2,297	(37)
Usuda town	2,156	1,941	(90)	1,922	(89)	742	(34)	743	(34)
Saku town	1,181	1,018	(86)	995	(84)	367	(31)	426	(36)
Yachiho village	668	607	(91)	600	(90)	259	(39)	265	(40)
Koumi town	849	733	(86)	723	(85)	288	(34)	289	(34)
Minami-aiki village	183	153	(84)	152	(83)	90	(49)	90	(49)
Kita-aiki village	124	113	(91)	111	(90)	60	(48)	60	(48)
Minamimaki village	422	379	(90)	379	(90)	156	(37)	157	(37)
Kawakami town	590	535	(91)	528	(89)	260	(44)	267	(45)
Ishikawa, Okinawa	7,309	5,217	(71)	5,156	(71)	800	(11)	814	(11)
Gushikawa city	6,281	4,420	(70)	4,359	(69)	443	( 7)	452	( 7)
Onna village	1,028	797	(78)	797	(78)	357	(35)	362	(35)
Sum of 4 population cohorts	27,063	20,665	(76)	20,266	(75)	6,532	(24)	6,556	(24)
Katsushika, Tokyo*	2,919	2,919	(100)	2,888	(99)	2,245	(77)	2,901	(99)
1990 participants	1,041	1,041	(100)	1,037	(100)	675	(65)	1,033	(99)
1991 participants	875	875	(100)	868	(99)	567	(65)	872	(100)
1992 participants	61	61	(100)	61	(100)	61	(100)	61	(100)
1993 participants	462	462	(100)	448	(97)	462	(100)	459	(99)
1994 participants	480	480	(100)	474	(99)	480	(99)	476	(99)
Total	29,982	23,584	(79)	23,154	(77)	8,777	(29)	9,457	(32)

* Participants of health check-up program at 40 and 50 years of age

**Table 1c.** Participants of baseline survey, cohort I females.

	Subjects	Questionnaire				Check-up data collected %		Blood provided %	

		returned %		analyzed %					
Ninohe, Iwate	6,269	5,117	(82)	4,872	(78)	3,267	(52)	3,276	(52)
Ninohe city	4,259	3,458	(81)	3,314	(78)	2,084	(49)	2,082	(49)
Karumai town	2,010	1,659	(83)	1,558	(78)	1,183	(59)	1,194	(59)
Yokote, Akita	8,223	6,426	(78)	6,283	(76)	3,432	(42)	3,007	(37)
Yokote city	6,484	4,753	(73)	4,649	(72)	2,403	(37)	2,107	(32)
Omonogawa town	1,739	1,673	(96)	1,634	(94)	1,029	(59)	900	(52)
Saku, Nagano	6,046	5,576	(92)	5,477	(91)	3,102	(51)	3,197	(53)
Usuda town	2,090	1,944	(93)	1,916	(92)	1,059	(51)	1,059	(51)
Saku town	1,157	1,034	(89)	1,016	(88)	467	(40)	540	(47)
Yachiho village	656	616	(94)	605	(92)	374	(57)	381	(58)
Koumi town	833	758	(91)	742	(89)	452	(54)	454	(55)
Minami-aiki village	199	186	(93)	182	(91)	117	(59)	118	(59)
Kita-aiki village	145	131	(90)	128	(88)	93	(64)	93	(64)
Minamimaki village	408	381	(93)	377	(92)	223	(55)	226	(55)
Kawakami town	558	526	(94)	511	(92)	317	(57)	326	(58)
Ishikawa, Okinawa	6,897	5,365	(78)	5,297	(77)	1,590	(23)	1,551	(22)
Gushikawa city	5,998	4,583	(76)	4,515	(75)	1,167	(19)	1,131	(19)
Onna village	899	782	(87)	782	(87)	423	(47)	420	(47)
Sum of 4 population cohorts	27,435	22,484	(82)	21,929	(80)	11,391	(42)	11,031	(40)
Katsushika, Tokyo*	4,178	4,177	(100)	4,116	(99)	3,143	(75)	4,149	(99)
1990 participants	1,399	1,399	(100)	1,386	(99)	880	(63)	1,390	(99)
1991 participants	1,336	1,335	(100)	1,325	(99)	821	(61)	1,334	(100)
1992 participants	112	112	(100)	109	(97)	112	(100)	109	(97)
1993 participants	571	571	(100)	545	(95)	570	(100)	563	(99)
1994 participants	760	760	(100)	751	(99)	760	(100)	753	(99)
Total	31,613	26,661	(84)	26,045	(82)	14,534	(46)	15,180	(48)

* Participants of health check-up program at 40 and 50 years of age

### Cohort II (Table 2a,b,c)

﻿﻿Among 62,398 population-based cohort subjects and 16,427 Suita cohort subjects, 52,256 (84%), 24,804 (81%) men and 27,452 (86%) women, and 10,960 (67%), 4,987 (62%) men and 5,973 (72%) women, returned their questionnaires, respectively. Although date of entry was distributed from January 1993 to December 1994 in 5 population-based cohorts, 67% was concentrated between February 1993 and March 1993 and the other subjects except subjects in Tomobe town, Kasama PHC area, responded within 1993. The baseline survey for 11,314 subjects in Tomobe town was conducted in 1994 and more than 99% responded in January or February in 1994. 18,894 (30%) population cohort, 6,582 (21%) men and 12,312 (39%) women, and 5,480 (33%) Suita cohort, 2,120 (26%) men and 3,360 (40%) women, provided their blood and stored their plasma and buffy layer at -80. We also obtained health checkup data from 19,292 (31%) population cohort, 6,476 (21%) men and 12,312 (40%) women, and 5,307 (32%) health checkup cohort, 1,993 (25%) men and 3,314 (40%) women.

**Table 2a.** Participants of baseline survey, cohort II.

	Subjects	Questionnaire				Check-up data collected %		Blood provided %	

		returned %		analyzed %					
Kasama, Ibaraki	21,488	19,490	(91)	19,012	(88)	5,626	(26)	5,244	(24)
Tomobe town	12,463	11,314	(91)	10,990	(88)	2,632	(21)	2,447	(20)
Iwase town	9,025	8,176	(91)	8,022	(89)	2,994	(33)	2,797	(31)
Kashiwazaki, Niigata									
Oguni town	3,571	3,236	(91)	3,165	(89)	875	(25)	1,116	(31)
Tosayamada, Kochi	8,606	7,737	(90)	7,557	(88)	2,641	(31)	2,624	(30)
Kagami town	2,596	2,426	(93)	2,411	(93)	801	(31)	800	(31)
Noichi town	6,010	5,311	(88)	5,146	(86)	1,840	(31)	1,824	(30)
Arikawa, Nagasaki	14,624	11,367	(78)	11,199	(77)	4,637	(32)	4,522	(31)
Uku town	1,999	1,457	(73)	1,429	(71)	741	(37)	742	(37)
Ojika town	2,000	1,717	(86)	1,704	(85)	973	(49)	880	(44)
Sin-uonome town	2,262	1,964	(87)	1,931	(85)	630	(28)	637	(28)
Arikawa town	3,455	2,863	(83)	2,833	(82)	904	(26)	836	(24)
Kamigoto town	3,076	1,959	(64)	1,936	(63)	833	(27)	868	(28)
Narao town	1,832	1,407	(77)	1,366	(75)	556	(30)	559	(31)
Miyako, Okinawa	14,109	10,426	(74)	10,048	(71)	5,513	(39)	5,388	(38)
Hirara city	10,891	7,770	(71)	7,516	(69)	3,702	(34)	3,607	(33)
Gusukube town	3,218	2,656	(83)	2,532	(79)	1,811	(56)	1,781	(55)
Sum of 5 population cohorts	62,398	52,256	(84)	50,981	(82)	19,292	(31)	18,894	(30)
Suita, Osaka	16,427	10,960	(67)	10,890	(66)	5,307	(32)	5,480	(33)
Suita1*	9,747	6,557	(67)	6,489	(67)	2,512	(26)	2,432	(25)
Suita2**	6,680	4,403	(66)	4,401	(66)	2,795	(42)	3,048	(46)
Total	78,825	63,216	(80)	61,871	(78)	24,599	(31)	24,374	(31)

* Subjects of health check-up program at 40 and 50 years of age, ** Randomly selected subjects

**Table 2b.** Participants of baseline survey, cohort II males.

	Subjects	Questionnaire				Check-up data collected %		Blood provided %	

		returned %		analyzed %					
Kasama, Ibaraki	10,777	9,636	(89)	9,453	(88)	1,787	(17)	1,703	(16)
Tomobe town	6,270	5,606	(89)	5,487	(88)	750	(12)	693	(11)
Iwase town	4,507	4,030	(89)	3,966	(88)	1,037	(23)	1,010	(22)
Kashiwazaki, Niigata									
Oguni town	1,774	1,558	(88)	1,520	(86)	295	(17)	441	(25)
Tosayamada, Kochi	4,120	3,614	(88)	3,561	(86)	920	(22)	919	(22)
Kagami town	1,244	1,140	(92)	1,140	(92)	290	(23)	290	(23)
Noichi town	2,876	2,474	(86)	2,421	(84)	630	(22)	629	(22)
Arikawa, Nagasaki	6,899	5,051	(73)	4,983	(72)	1,303	(19)	1,270	(18)
Uku town	916	638	(70)	625	(68)	205	(22)	206	(22)
Ojika town	952	783	(82)	780	(82)	377	(40)	344	(36)
Sin-uonome town	1,041	846	(81)	831	(80)	136	(13)	142	(14)
Arikawa town	1,614	1,275	(79)	1,261	(78)	249	(15)	208	(13)
Kamigoto town	1,492	868	(58)	861	(58)	186	(12)	217	(15)
Narao town	884	641	(73)	625	(71)	150	(17)	153	(17)
Miyako, Okinawa	7,081	4,945	(70)	4,779	(67)	2,171	(31)	2,249	(32)
Hirara city	5,426	3,642	(67)	3,537	(65)	1,401	(26)	1,469	(27)
Gusukube town	1,655	1,303	(79)	1,242	(75)	770	(47)	780	(47)
Sum of 5 population cohorts	30,651	24,804	(81)	24,296	(79)	6,476	(21)	6,582	(21)
Suita, Osaka	8,089	4,987	(62)	4,962	(61)	1,993	(25)	2,120	(26)
Suita1*	4,793	2,931	(61)	2,907	(61)	749	(16)	729	(15)
Suita2**	3,296	2,056	(62)	2,055	(62)	1,244	(38)	1,391	(42)
Total	38,740	29,791	(77)	29,258	(76)	8,469	(22)	8,702	(23)

* Subjects of health check-up program at 40 and 50 years of age, ** Randomly selected subjects

**Table 2c.** Participants of baseline survey, cohort II females.

	Subjects	Questionnaire				Check-up data collected %		Blood provided %	

		returned %		analyzed %					
Kasama, Ibaraki	10,711	9,854	(92)	9,559	(89)	3,839	(36)	3,541	(33)
Tomobe town	6,193	5,708	(92)	5,503	(89)	1,882	(30)	1,754	(28)
Iwase town	4,518	4,146	(92)	4,056	(90)	1,957	(43)	1,787	(40)
Kashiwazaki, Niigata									
Oguni town	1,797	1,678	(93)	1,645	(92)	580	(32)	675	(38)
Tosayamada, Kochi	4,486	4,123	(92)	3,996	(89)	1,721	(38)	1,705	(38)
Kagami town	1,352	1,286	(95)	1,271	(94)	511	(38)	510	(38)
Noichi town	3,134	2,837	(91)	2,725	(87)	1,210	(39)	1,195	(38)
Arikawa, Nagasaki	7,725	6,316	(82)	6,216	(81)	3,334	(43)	3,252	(42)
Uku town	1,083	819	(76)	804	(74)	536	(49)	536	(49)
Ojika town	1,048	934	(89)	924	(88)	596	(57)	536	(51)
Sin-uonome town	1,221	1,118	(92)	1,100	(90)	494	(40)	495	(41)
Arikawa town	1,841	1,588	(86)	1,572	(85)	655	(36)	628	(34)
Kamigoto town	1,584	1,091	(69)	1,075	(68)	647	(41)	651	(41)
Narao town	948	766	(81)	741	(78)	406	(43)	406	(43)
Miyako, Okinawa	7,028	5,481	(78)	5,269	(75)	3,342	(48)	3,139	(45)
Hirara city	5,465	4,128	(76)	3,979	(73)	2,301	(42)	2,138	(39)
Gusukube town	1,563	1,353	(87)	1,290	(83)	1,041	(67)	1,001	(64)
Sum of 5 population cohorts	31,747	27,452	(86)	26,685	(84)	12,816	(40)	12,312	(39)
Suita, Osaka	8,338	5,973	(72)	5,928	(71)	3,314	(40)	3,360	(40)
Suita1*	4,954	3,626	(73)	3,582	(72)	1,763	(36)	1,703	(34)
Suita2**	3,384	2,347	(69)	2,346	(69)	1,551	(46)	1,657	(49)
Total	40,085	33,425	(83)	32,613	(81)	16,130	(40)	15,672	(39)

* Subjects of health check-up program at 40 and 50 years of age, ** Randomly selected subjects

## DISCUSSIONS

The JPHC Study includes 29 study sub-areas (city, town or village level) from 11 PHC area. These sub-areas are divided into 3 types: urban city with over 300,000 populations (Katsushika ward and Suita city), local city with over 20,000 populations (Ninohe, Yokote, Gushikawa and Hirara cities), rural town or village (the other 23 towns or villages). The questionnaire and the collection of blood and checkup data were applied only for participants of health checkup program in Katsushika area and therefore the response rate was substantially higher than the other areas. Although the method applied in two sub-areas in Suita city were slightly different from population-based cohort, the subjects were both extracted from population registry and not participants of health checkup program. The response rates to the questionnaire tended to be higher (87%) in 23 rural town or villages, lower (66%) in urban city (Suita city) and intermediate (73%) in 4 local cities. The same trend was also observed for the collection rates of blood and checkup data which were only collected from the participants of local healthcheck up program, although the rates were lower in a Gushikawa city when compared with other local cities.

The response rates were consistently lower in men than in women. The sexual differences were more marked in the collection rates of blood and checkup data. This is because women tend to participate in health checkup program provided by local government which is our major source of checkup data and blood collections. Men usually take a health checkup in their workplace.

In summary, among 140,420 targeted subjects, we created a database with information from 113,461 (81%) questionnaires regarding lifestyle and from 47,910 (34%) health checkup data. We also stored 3 aliquotes of plasma and one aliquote of buffy coat from 49,011 subjects. These data and blood samples serve as basis for JPHC Study.
